# Tapia’s Syndrome Secondary to Prolonged Intubation and Tracheostomy in a Patient With Opioid Dependence With Spontaneous Recovery

**DOI:** 10.7759/cureus.83414

**Published:** 2025-05-03

**Authors:** Portia N Mzezewa, Sohaib Eladl, Muhammad Nasser Javaid, Mohamed F Ahmed, Laurat H Zoaka

**Affiliations:** 1 Respiratory Medicine, Blackpool Teaching Hospitals National Health Service (NHS) Foundation Trust, Blackpool, GBR; 2 Accident and Emergency, Blackpool Teaching Hospitals National Health Service (NHS) Foundation Trust, Blackpool, GBR; 3 Diabetes and Endocrinology, Blackpool Teaching Hospitals National Health Service (NHS) Foundation Trust, Blackpool, GBR

**Keywords:** opiod abuse, percutaneous tracheostomy procedures, tapia's syndrome, tongue deviation, transoral intubation

## Abstract

Tapia syndrome is known as a rare complication characterized by neurologic deficits involving the hypoglossal nerve (XII) and recurrent laryngeal branch of the vagal nerve (X). It can occur after airway manipulation, including intubation. Typical signs and symptoms include dysphagia and dysphonia. This case report describes a case of Tapia syndrome post-intubation in a critical care unit. He is a 46-year-old male with a background of opioid dependence who presented with a reduced level of consciousness and type 1 respiratory failure (T1RF). He had multiple intubations during his hospital stay and prolonged mechanical ventilation requiring a tracheostomy. He developed ventilator-associated pneumonia and *C. difficile* infection. After decannulation, he developed persistent dysphonia, dysphagia, tongue deviation, and aspiration. He had a nasendoscopy, which revealed a paralyzed right vocal cord in the paramedian position and tongue deviation, which is consistent with Tapia's syndrome. Initially, he was considered for injection laryngoplasty, but he showed significant spontaneous improvement as his left vocal cord was compensating very well. Despite being recorded before in the literature, our case is important to recognize Tapia's syndrome post-intubation and the challenges of managing airway complications in patients after opioid dependence. It also highlights the potential for spontaneous recovery with conservative management in Tapia's syndrome, as this spontaneous recovery is not well-documented in the literature, and most of the cases require steroids, vitamin B complex, or even more invasive interventions to improve the condition.

## Introduction

Tapia's syndrome is a rare condition first described by Antonio Garcia Tapia in 1904, and from here comes the name. An article by Cariati P, Cabello A, Galvez PP, et al. (2016) in the Journal of Medical Case Reports highlighted fewer than 100 cases having been described in the literature at that time [1]. Tapia syndrome is a classic triad of ipsilateral vocal cord paralysis, tongue deviation, and dysphagia, typically caused by extracranial injury to these nerves. When the nerves course near the carotid artery and hyoid bone, it becomes liable to injury. While most commonly reported after airway manipulation procedures, it can also occur secondary to vascular lesions, trauma (post-intubation), or tumours. Looking at the literature, a case of Tapia syndrome by Lim KJ, Kim MH, Kang MH, et al. in the Korean J Anaesthesiology Journal (2013) highlighted it following cervical laminoplasty and general anaesthesia with uncomplicated endotracheal intubation, where they treated it with videofluoroscopic swallowing study (VFSS)-guided balloon dilation of the oesophagus [2].

## Case presentation

**Figure 1:**
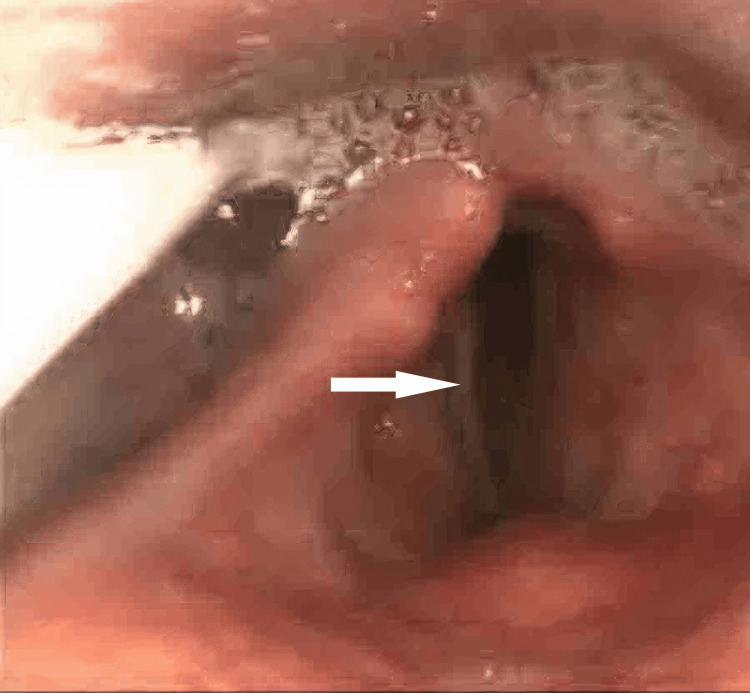
Flexible nasendoscopy (FNE) showing Rt. vocal cord paralysis

**Figure 2:**
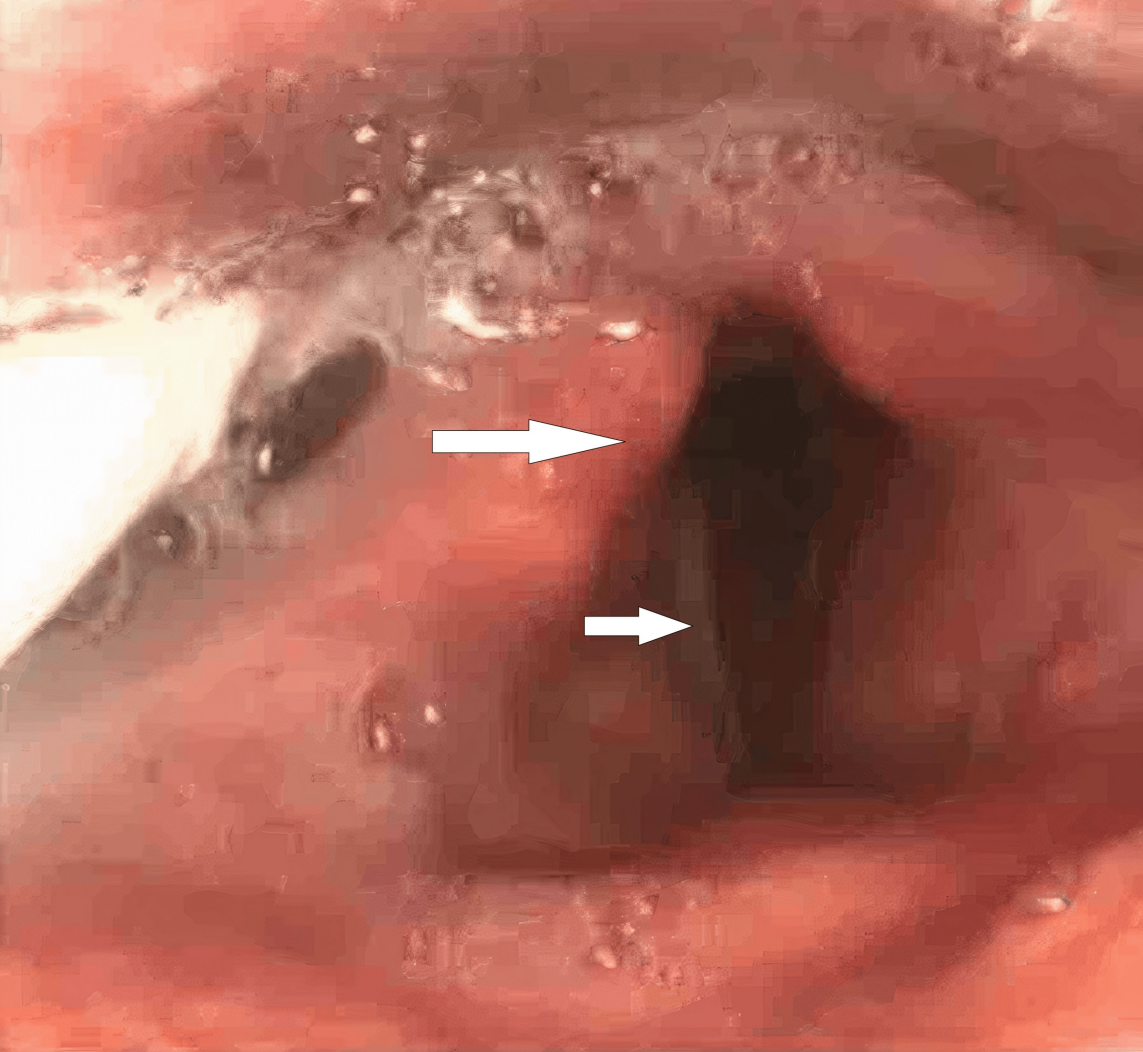
Flexible nasendoscopy (FNE) showing Rt. vocal cord paralysis with bowing

**Figure 3:**
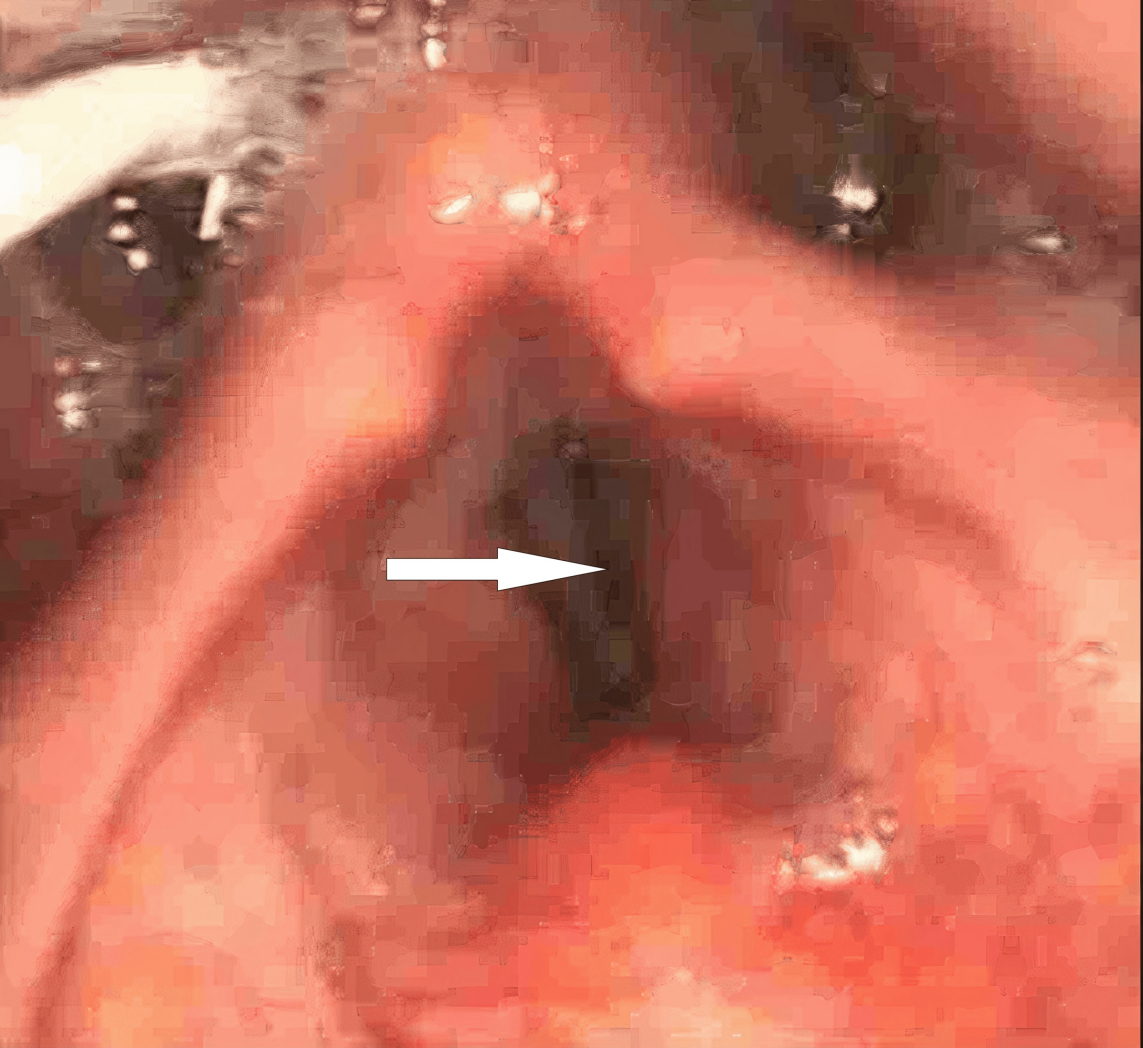
Flexible nasendoscopy (FNE) showing phonatory gap

A 46-year-old male with a significant history of opioid dependence (currently on opioid blocker therapy). His past medical history included asthma, hypertension and obstructive sleep apnoea (OSA) with home continuous positive airway pressure (CPAP). He presented to the emergency department in October 2024 with type 1 respiratory failure (T1RF) and a reduced level of consciousness following a presumed recreational drug overdose. Endotracheal intubation and mechanical ventilation were required to manage him initially in the intensive care unit (ICU), as he went straight forward from the emergency department (ED) to the ICU, where he stayed for 19 days. His hospital stay course was complicated by multiple self-extubations due to agitation and difficulty with sedation, as he was already on opioid blocker therapy. Consequently, he required re-intubation on several occasions, with very noticeable difficult airway management. He underwent percutaneous tracheostomy due to prolonged ventilator dependence. After being on the ventilator for a while, he developed ventilator-associated pneumonia, as resistant Klebsiella and C. difficile colitis were also identified during his stay in the intensive care unit. Eight days later, and after 19 days in critical care, he was successfully decannulated. He was stepped down to a respiratory ward to take care of his pneumonia and C. difficile colitis he developed. Post-extubation, he started developing persistent hoarseness and weak voice production. He had significant difficulty in swallowing (dysphagia) with recurrent episodes of aspiration. He had a right tongue and palatal deviation, with his tongue movement being reduced bilaterally. He had significant copious secretions production, and for that, he required nasogastric tube feeding. Input from the neurology team was requested, and his neurological examination showed palsy of the right hypoglossal and recurrent laryngeal nerves, with the rest of the cranial nerve examination being normal. Magnetic resonance neck imaging showed an area of abnormal soft tissue thickening noted at the right side of the oropharynx, centred on the palatine tonsil with extension into the right-sided base of the tongue and some extension into the right-sided supraglottic/glottic space. Abnormal thickening of the right-sided vocal cord. This lesion was noted to be inseparable from the right-sided epiglottis. Obliteration of the right-sided vallecula and subsequent narrowing of the right oropharynx were noted. The radiologist suggested direct visualisation via flexible nasendoscopy (FNE), which was done later by the ENT team. No evidence of acute cerebrovascular diseases was detected in the scan. His CT head and neck only showed some chest infective changes, with his blood being unremarkable. An ENT evaluation was requested for him as an inpatient, and he had an initial nasendoscopy, which revealed complete paralysis of his right vocal cord in a paramedian position accompanied by good compensatory movement of the left one (Figures 1, 2). A significant glottic gap (phonatory gap) was identified, and he was considered for injection laryngoplasty in a follow-up appointment three weeks later (Figure 3). That follow-up evaluation showed subjective improvement in his voice quality with persistent right vocal cord paralysis, but the left cord compensation was better. His consideration for injection laryngoplasty was deferred due to clinical improvement. A multidisciplinary approach involving the ear, nose, and throat (ENT), ICU, and speech and language therapy (SALT) team was started for him, considering nasogastric (NG) tube feeding initially, with discussion of potential percutaneous endoscopic gastrostomy (PEG) placement. He had some voice therapy and swallowing rehabilitation with a planned three-month ENT follow-up to assess progress, which seems to be heading in the right direction, not requiring more than a conservative management approach.

## Discussion

The combined injury to cranial nerves X and XII in Tapia's syndrome typically occurs where these nerves are in close proximity to the carotid bifurcation and hyoid bone. In our case, several mechanisms likely contributed to this, as it could have happened due to direct compression from endotracheal tube cuff inflation, a stretching injury during these multiple intubations and extubations or, most likely, trauma from the tracheostomy procedure itself. Due to the prolonged positioning of the patient, potential ischaemic injury is one of the significant considerations. In our case, the patient's opioid dependence and opioid blocker therapy created additional challenges, as this increased the risk of self-extubation due to the inability to adequately sedate him, leading to multiple re-intubation attempts, which obviously increased the trauma risk and the potential for more forceful airway manipulation. For this case, several diagnostic considerations were documented, including Tapia's syndrome (combined X and XII), isolated recurrent laryngeal nerve injury or even central causes (e.g., brainstem stroke). The multidisciplinary approach followed involving neurology and ENT team investigations specifically deemed this case as a post-intubation Tapia syndrome. The management approach for this case highlights several important principles, including initial stabilisation, diagnostic workup, therapeutic options and monitoring progression. For the initial stabilisation, airway protection post-extubation was one of the challenges, and nutritional support via NG tube was a considerable challenge. Diagnostic workup-wise, nasendoscopy as a primary diagnostic tool was used to visualise the movement of the cords and considerations of imaging (computed tomography/magnetic resonance imaging [CT/MRI]) if progression happened or atypical features started developing. The therapeutic options included voice therapy, swallowing rehabilitation and surgical options (injection laryngoplasty) but were deferred given the improvement recorded. To monitor progression, serial clinical assessments and objective voice measurements have been planned for him. What makes this one a unique case of Tapia syndrome is the complex medical background with opioid dependence, multiple intubation events increasing injury risk and the followed multidisciplinary management approach. The recorded significant spontaneous improvement despite initial severe symptoms was something not very well documented in the literature. Looking at the literature, this case had more complex precipitating factors (multiple intubations, tracheostomy); however, still, conservative management, including speech and language therapy along with swallowing rehabilitation, played a key role in improvement.

In 2013, the Laryngoscope Journal reported a case by Gevorgyan A, Nedzelski JM with a late diagnosis (three years post-injury) after a suspected airway manipulation where they used empiric corticosteroids and dysphagia therapy. The same article also highlighted recovery of Tapia syndrome being excellent in 30% of patients, incomplete in 39% of patients, and none in over 26% of patients [3]. Again in 2012, Tapia's syndrome was reported as an unexpected but real complication of rhinoplasty in the Aesthetic Plastic Surgery Journal by Lykoudis EG and Seretis K, where a short course of systemic steroids and vitamin B complex was practised [4]. Brotis AG, Hajiioannou J, Tzerefos C, et al. reported in the Br J Neurosurg Journal (2019) bilateral Tapia's syndrome secondary to cervical spine injury in a 24-year-old man where treatment with steroids was initiated [5]. Again in 2013, Varedi P, Shirani G, Karimi A, et al. in the Journal of Oral Maxillofacial Surgery reported Tapia syndrome in a 27-year-old man after repairing a fractured zygomatic complex, and in that case, the patient underwent nonsurgical management with an oral steroid (tablet of prednisolone, 60 mg/d, which was tapered down over 14 days) and vitamin B complex, together with speech and swallowing therapy (for almost two months) [6]. In 2022, Steehler AJ, Rothman R, Sadhar B, et al. reported Tapia's Syndrome after Cardiac Surgery in Ear Nose Throat Journal. The patient was a 70-year-old male who underwent aortic valve replacement (AVR) and coronary artery bypass grafting (CABG). He was placed in the supine position, was administered general anaesthesia, and was intubated orotracheally. A decision was made for the vocal cord injection with a carboxymethylcellulose gel implant, which occurred one week later (on postoperative day 36). The patient was injected with 1.0 cm³ of the implant into the left vocal cord at the level of the vocal process and just anterior to the superior arcuate line [7]. In the Cureus Journal (2021), Stelman CR, Buxton W, and Sharon JD reported Tapia's Syndrome following left retro sigmoid craniotomy for schwannoma resection when he later underwent a right vocal fold injection with Prolaryn gel via flexible laryngoscopy with a slight improvement in his dysphonia [8]. Tapia Syndrome after cervical laminoplasty, a posterior approach in a flexed head position was reported in the World Neurosurgery Journal in 2020 by Waits KD, Kelman CR, and Cameron BM [9]. Tapia syndrome following orotracheal intubation was reported in 2018 by Silva-Hernández L, Gil Rojo C, et al. in Neurologia (Engl Ed) [10]. Tapia's syndrome after cosmetic malar augmentation (Medpor implant) with transnasal intubation was mentioned in the literature in 2019 in the Journal of Dentistry, Shiraz University, by Ghorbani F, Tavanafar S, and Eftekharian H [11]. The patient was treated immediately after the diagnosis with 0.5mg dexamethasone for two weeks. After three months, the movements of the vocal cord and tongue movement started to improve, and the patient’s hoarseness fully recovered after six months [11]. In 2002, Tapia syndrome post-shoulder surgery (arthroscopy) in a sitting position with head lateral flexion was reported in the British Journal of Anaesthesia by Boisseau N, Rabarijaona H, Grimaud D, et al., when corticosteroid therapy and vitamins B1 and B6 were given for two weeks and speech therapy started [12]. In 2007, the European Journal of Anaesthesiology published postoperative airway obstruction due to Tapia's syndrome after coronary bypass grafting surgery [13]. In 2012, Tapia's syndrome as a rare complication following cardiac surgery was reported in the Journal of the European Association for Cardio-Thoracic Surgery Post-CABG with general anaesthesia and the patient was empirically treated with steroids (dexamethasone 4 mg i.v. thrice a day) [14].

In 2023, Piagnerelli M, Ranwez D, Arnould P, et al. reported Tapia syndrome as an unusual complication to recognise at the time of the COVID-19 pandemic in the Archives of Clinical and Medical Case Reports, where they reported two cases (one unilateral, one bilateral) [15]. In the National Journal of Maxillofacial Surgery (2023), Tapia syndrome was reported in a 17-year-old patient who developed Tapia syndrome following temporomandibular joint (TMJ) gap arthroplasty for ankylotic release of the left temporomandibular joint [16]. Several other cases have reported the use of steroids as part of the management of Tapia syndrome, and these include a case of a 49-year-old patient with Tapia’s syndrome in the intensive care unit following intubation reported by M. Coninckx (2015) in Acta Neurologica Belgica [17]. In 1983, the JAMA Otolaryngology-Head & Neck Surgery Journal published a case of Tapia's syndrome after thoracotomy [18].

## Conclusions

This particular case of Tapia's syndrome raises risk factor awareness, as patients requiring multiple intubations, especially those with challenging airways due to conditions like opioid dependence, are at increased risk for cranial nerve injuries. Patients presenting with voice changes and swallowing difficulties post-intubation raise the consideration for it. The initial management for these cases should be a conservative approach for post-intubation vocal cord paralysis, with surgical interventions considered only if no improvement occurs after adequate time for recovery.

The significant spontaneous improvement in this patient suggests that a period of observation is required in similar cases. While Tapia's syndrome typically has a good prognosis, each case must be individualised based on the extent of nerve injury and patient-specific factors like comorbidities.
